# Supplementary material for: Modeling glioblastoma heterogeneity as a dynamic network of cell states
Source: Mol Syst Biol. 2021 Sep 16;17(9):e10105. doi: 10.15252/msb.202010105 (PMC8444284; doi:10.15252/msb.202010105)
Supplement: Supplementary file 6 — Source Data for Figure 5 [file MSB-17-e10105-s004.zip › Figure5A_sourcedata/GSEA_3071/hallmarks_stateI.GseaPreranked.1623742531165/HALLMARK_ADIPOGENESIS.html]

Details for gene set HALLMARK\_ADIPOGENESIS[GSEA]

|  || Dataset | state13071 |
| Phenotype | NoPhenotypeAvailable |
| Upregulated in class | na\_pos |
| GeneSet | HALLMARK\_ADIPOGENESIS |
| Enrichment Score (ES) | 0.46417478 |
| Normalized Enrichment Score (NES) | 2.3715587 |
| Nominal p-value | 0.0 |
| FDR q-value | 2.952381E-4 |
| FWER p-Value | 0.001 |
Table: GSEA Results Summary

  

Fig 1: Enrichment plot: HALLMARK\_ADIPOGENESIS      
 Profile of the Running ES Score & Positions of GeneSet Members on the Rank Ordered List

  

| PROBE | GENE SYMBOL | GENE\_TITLE | RANK IN GENE LIST | RANK METRIC SCORE | RUNNING ES | CORE ENRICHMENT || 1 | SSPN |  |  | 23 | 1.574 | 0.0414 | Yes |
| 2 | COL15A1 |  |  | 25 | 1.550 | 0.0885 | Yes |
| 3 | COL4A1 |  |  | 28 | 1.524 | 0.1344 | Yes |
| 4 | ITIH5 |  |  | 35 | 1.473 | 0.1777 | Yes |
| 5 | ENPP2 |  |  | 55 | 1.330 | 0.2128 | Yes |
| 6 | APOE |  |  | 60 | 1.294 | 0.2512 | Yes |
| 7 | OMD |  |  | 75 | 1.196 | 0.2836 | Yes |
| 8 | NABP1 |  |  | 154 | 0.907 | 0.2888 | Yes |
| 9 | LAMA4 |  |  | 176 | 0.863 | 0.3091 | Yes |
| 10 | SLC1A5 |  |  | 199 | 0.831 | 0.3281 | Yes |
| 11 | CHCHD10 |  |  | 225 | 0.778 | 0.3446 | Yes |
| 12 | ANGPT1 |  |  | 289 | 0.689 | 0.3474 | Yes |
| 13 | PDCD4 |  |  | 295 | 0.682 | 0.3668 | Yes |
| 14 | CMBL |  |  | 311 | 0.662 | 0.3827 | Yes |
| 15 | ABCA1 |  |  | 345 | 0.633 | 0.3925 | Yes |
| 16 | DHRS7 |  |  | 398 | 0.589 | 0.3954 | Yes |
| 17 | MGLL |  |  | 406 | 0.581 | 0.4111 | Yes |
| 18 | IFNGR1 |  |  | 407 | 0.580 | 0.4289 | Yes |
| 19 | TALDO1 |  |  | 455 | 0.545 | 0.4319 | Yes |
| 20 | TANK |  |  | 464 | 0.538 | 0.4460 | Yes |
| 21 | POR |  |  | 515 | 0.499 | 0.4468 | Yes |
| 22 | ME1 |  |  | 543 | 0.484 | 0.4538 | Yes |
| 23 | MGST3 |  |  | 586 | 0.460 | 0.4557 | Yes |
| 24 | DNAJB9 |  |  | 605 | 0.448 | 0.4642 | Yes |
| 25 | SCP2 |  |  | 868 | 0.361 | 0.3994 | No |
| 26 | REEP5 |  |  | 881 | 0.355 | 0.4068 | No |
| 27 | LIFR |  |  | 904 | 0.350 | 0.4111 | No |
| 28 | PHLDB1 |  |  | 1061 | 0.315 | 0.3757 | No |
| 29 | RAB34 |  |  | 1137 | 0.301 | 0.3632 | No |
| 30 | ARL4A |  |  | 1195 | 0.289 | 0.3555 | No |
| 31 | MYLK |  |  | 1204 | 0.287 | 0.3620 | No |
| 32 | ALDH2 |  |  | 1321 | 0.265 | 0.3365 | No |
| 33 | UQCRQ |  |  | 1464 | -0.254 | 0.3032 | No |
| 34 | CCNG2 |  |  | 1610 | -0.269 | 0.2695 | No |
| 35 | NDUFAB1 |  |  | 1635 | -0.272 | 0.2709 | No |
| 36 | TST |  |  | 1779 | -0.291 | 0.2384 | No |
| 37 | MRPL15 |  |  | 1864 | -0.300 | 0.2233 | No |
| 38 | MTCH2 |  |  | 1924 | -0.308 | 0.2156 | No |
| 39 | IDH3A |  |  | 2020 | -0.320 | 0.1980 | No |
| 40 | SORBS1 |  |  | 2092 | -0.333 | 0.1876 | No |
| 41 | CYC1 |  |  | 2208 | -0.354 | 0.1652 | No |
| 42 | CAVIN2 |  |  | 2237 | -0.360 | 0.1681 | No |
| 43 | SCARB1 |  |  | 2273 | -0.366 | 0.1692 | No |
| 44 | QDPR |  |  | 2338 | -0.382 | 0.1623 | No |
| 45 | AGPAT3 |  |  | 2387 | -0.390 | 0.1604 | No |
| 46 | UCP2 |  |  | 2498 | -0.413 | 0.1412 | No |
| 47 | UQCR11 |  |  | 2614 | -0.443 | 0.1215 | No |
| 48 | COX8A |  |  | 2819 | -0.509 | 0.0780 | No |
| 49 | CDKN2C |  |  | 2903 | -0.544 | 0.0707 | No |
| 50 | APLP2 |  |  | 3034 | -0.621 | 0.0520 | No |
| 51 | GPX4 |  |  | 3103 | -0.679 | 0.0531 | No |
| 52 | ELOVL6 |  |  | 3168 | -0.748 | 0.0575 | No |
| 53 | ITGA7 |  |  | 3430 | -1.355 | 0.0234 | No |
Table: GSEA details [plain text format]

  

Fig 2: HALLMARK\_ADIPOGENESIS: Random ES distribution      
 Gene set null distribution of ES for **HALLMARK\_ADIPOGENESIS**
